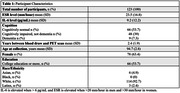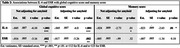# Serological inflammation markers and their association with cognition in the oldest old; The 90+ Study

**DOI:** 10.1002/alz.089918

**Published:** 2025-01-03

**Authors:** Anne‐Marie C Leiby, Ghasem Farahmand, Aanan Ramanathan, Davis C. Woodworth, Luohua Jiang, Jiaxin Yu, Tianchen Qian, María M. M. Corrada, Claudia H. Kawas, S. Ahmad Sajjadi

**Affiliations:** ^1^ University of California, Irvine, CA USA; ^2^ University of California, Irvine, Irvine, CA USA; ^3^ University of California Irvine, Irvine, CA USA

## Abstract

**Background:**

Recent studies in the younger old have identified associations between cognitive performance and markers of inflammation, including interleukin‐6 (IL‐6) and erythrocyte sedimentation rate (ESR). These associations remain unexplored in the oldest old (age 90+), an age group most vulnerable to dementia. In addition, no studies have examined if amyloid burden impacts these associations. This study aims to: (1) examine the associations between inflammatory markers (IL‐6, ESR) and cognitive performance in individuals age 90+ and (2) to examine if amyloid burden impacts these associations.

**Method:**

Participants with at least one plasma measure of inflammation and amyloid burden measured by positron emission tomography (PET) were selected from ***The 90+ Study*** (n = 112 for IL‐6, n = 123 for ESR). Cognitive measures included (1) global cognitive score defined as the average of the standardized scores of Mini‐Mental State Examination (MMSE) and Modified MMSE (3MS), and (2) memory score, that was the average of the standardized scores of California Verbal Learning Test (CVLT) and memory items of 3MS. PET amyloid burden was measured by standardized uptake value ratio (SUVR). We examined the associations between IL‐6 and ESR (independent variables) with cognitive measures (dependent variable) using linear regression models. Two models were implemented: first, without adjustment and second, with adjustment for amyloid. Both models were also adjusted for age at blood draw, sex, and education (college/no college). We additionally adjusted for amyloid burden and years between blood draw and PET scan in the second model.

**Result:**

Mean age at blood draw was 94.7±2.8 and most participants were female (63.4%) and white (92.7%) (Table 1). In model 1, for both IL‐6 and ESR, higher levels were significantly associated with lower global cognitive scores (Table 2). Only IL‐6 was significantly associated with memory score (Table 2). Amyloid adjustment in model 2 had little effect on our results (Table 2).

**Conclusion:**

This study suggests that systemic inflammation could contribute to cognitive performance in individuals aged 90 and older. Additionally, this influence may be linked to a process not attributed to amyloid. Further research is warranted to explore the role of inflammation in non‐Alzheimer’s disease dementias.